# Supplementary material for: An open-source perceptual crossing device for investigating brain dynamics during human interaction
Source: PLoS One. 2024 Jun 10;19(6):e0305283. doi: 10.1371/journal.pone.0305283 (PMC11164400; doi:10.1371/journal.pone.0305283)
Supplement: Attachment — Submitted filename: Response to Reviewers.pdf [file pone.0305283.s001.pdf]

Response to Reviewers

To the academic editor and reviewer,

Thank you for your thorough review and insightful feedback on the Perceptual Crossing Device (PCD) technical paper. We greatly appreciate the reviewer's assessment of the engineering aspects as being "*an excellent example of engineering innovation applied to address critical and difficult questions in the social sciences*". We have carefully considered all your comments and points raised and have addressed them below. Once again, thank you for your valuable feedback.

Response to Editor

E1) *"In the motivation of the study, it is reported that previous devices were limited at the location and area of vibrostimulation (lines 53-55). I recommend making clearer the reasons as to why this can be considered a limitation."*

This was identified as a limitation because a smaller area of vibration makes the devices less engaging. In the current model, by utilizing both hands and expanding the area of stimulation, we enhance user feedback significantly, which is crucial for maintaining attention — the more pronounced the experience, the more engaged the user. To do so, we updated the following in the manuscript:

"The PCD presented here offers tactile feedback for both hands through the moving handle and the button-pressing handle. Employing both hands to enlarge the area of vibration enhances the stimulation, rendering it more engaging and providing a pronounced experience pertinent to social modalities." (Line 68-71)

E2) *"Line 230, there is a reference to a 'marker', but it is unclear what marker is being referred to."*

In this context, 'marker' refers to a timestamp to precisely indicate the occurrence of events during data collection. This is a crucial feature of our implementation based on integration with Brain Products' trigger box. This has been properly revised in the lines below:

"To ensure the accurate integration of various components of the PCD, the Brain Products STIMTRAK Acoustical Stimulator Adapter was employed, chosen for its noted high precision in

event marker generation. A marker acts as a timestamp that shows the exact time when events occurred in line with the EEG data." (Line 263 - 266)

<u>Response to Reviewer</u>

Major Comments

R1) *"How do the authors think the PCD contributes to this rich and ongoing dialogue? Does the PCD create opportunities for novel experimental designs or analysis? Does the PCD create opportunities for novel experimental designs or analysis?"*

The PCD addresses this by being an open-source and reprogrammable tool, the first time a PCD has been made available in the public domain for replication. This design gives researchers working in the social domain of haptics, the ability to download, rebuild, and customize both the device and its software to meet their specific research needs. Such flexibility is anticipated to spur innovative experiments and analyses as the field progresses. We have added the following sentences to clarify this opportunity:

"Ultimately, the PCD leverages open-source software customization and replicable hardware design to foster innovation in experimental methodologies and data analysis within its field. This flexibility allows researchers to modify the code to suit their unique experimental needs, enabling the generation of innovative insights that are reproducible using the same open-source equipment." (Line 377 – 381)

Moreover, the PCD contributes to the existing dialogue by incorporating features utilized in previous experiments, such as vibration and sound. For example, audio feedback, which was used in past experiments by Lenay and Stewart (2012), is also integrated into the PCD. By combining this flexibility with capabilities from prior research, the PCD not only supports the continuation and growth of existing experimental frameworks but also opens new avenues for conducting future experiments.

Lenay C, Stewart J. Minimalist approach to perceptual interactions. Frontiers in Human Neuroscience. 2012;6:98.

R2) *"The authors say 'The biggest drawback of previous iterations is the lack of an immediate feedback for the user of their actual movements in the non-visible space' (lines 48-49).*

Could the authors motivate this claim more? I'd like to understand why the authors think this particular complication will benefit investigations of social interaction, beyond being an interesting engineering innovation. "

We would like to emphasize the innovation introduced in the current version of the PCD. One of the most significant limitations identified in prior versions was the absence of immediate 1-to-1 feedback for the user regarding their movements in the non-visible space, also demonstrated in Maeda et al. (2011). The latest iteration of the PCD addresses this issue by providing 1-to-1 immediate feedback to the user, described more in the sentence below:

"This 1-to-1 immediate feedback is significant because it focuses on the social presence and social interaction between people, not the constitution of space and how they understand space." (Line 64-66)

This feature is vital as it enhances the investigation of social interactions by ensuring users can immediately perceive and respond to changes in their environment. This focus on immediate feedback is designed not merely to explore the spatial awareness of the participants but primarily to enhance the quality of social presence and interaction among them. This directly contributes to a more authentic and engaging user experience, which is central to the objectives of our research. Additionally, researchers interested in investigating the condition under which virtual experience is constituted could change this 1-to-1 mapping to an alternative mapping.

Maeda T, Ando H, Iizuka H, Yonemura T, Kondo D, Niwa M. Parasitic Humanoid: the wearable robotics as a behavioral assist interface like oneness between horse and rider. In: Proceedings of the 2nd Augmented Human International Conference. AH '11. New York, NY, USA: Association for Computing Machinery; 2011.Available from: https://doi.org/10.1145/1959826.1959844.

R3) *"While PLOS ONE is largely a generalist journal, the intended audience for this manuscript is unclear to me. If the authors have a more engineering-oriented audience in mind, then I would expect additional explication of the PCE, its contribution to the study of social interaction, and more details about previous PCDs. If the idea audience is psychologists studying social interaction, then it seems necessary to me to report whether the modifications to this PCD (e.g. providing access to spatial information to participants) influence behavior in any way. In either case, I would like to see at least a brief discussion of how the modifications and additions to this version of PCD will contribute to the literature on PCE specifically and social interaction generally. If the authors are aiming for a multidisciplinary reach, then all of these items are important."*

We now clarify who our primary audience comprises of with the following additional sentences:

"Furthermore, unlike earlier versions of the PCD, details on validation and replicability have not been publicly disclosed until now. We are the first to provide such information, offering valuable resources for researchers, behavioral scientists, cognitive scientists, cognitive neuroscientists, and psychologists." (Line 84 – 87)

"The introduction of the Perceptual Crossing Device (PCD) in this study establishes a novel framework aimed at supplying a transparent, publicly available device for researchers working with haptics interested in the social domain to examine sensorimotor dyadic interactions in real-time." (Line 24 – 28)

We have demonstrated that despite the modifications introduced in the PCD, we can successfully replicate findings from previous studies, as shown in Lerique et al. (2024). This indicates that the changes to the device have not negatively affected behavioral outcomes. Furthermore, these modifications have enriched the literature by demonstrating the robustness of our findings across different experimental setups. We have thereby provided a platform that enhances reproducibility and flexibility in research methodologies. This alignment with the needs of our target audience ensures that the PCD can promote the PCE as a valuable tool for advancing research in social interaction.

Lerique S, Estelle S, Hayashi SR, Leenen I, Morrissey BR, Morrissey T, et al.. The ECSU-PCE Dataset: A comprehensive recording of embodied social interaction with EEG, peripheral physiology, and behavioral measurements in adults; 2024. Available from: osf.io/6hjfy.

R4) *"The authors note several technical challenges for those without specific engineering skills. How accessible are, for example, online tutorials (e.g. YouTube) for acquiring the necessary skills to implement the PCD?"*

Regarding the assembly of the PCD, we now mention the expectations regarding the skill level required.

"Key difficulties encountered in the assembly process include soldering, wire preparation, threading of 3D printed components, and software installation. However, the expectation is that the skill level of an undergraduate engineering intern would have the capability of assembling the entire PCD setup within 1 week." (Line 97 – 101)

The essential skills required for assembling the PCD, such as soldering and thread tapping, are basic and can be swiftly learned through numerous free online resources (e.g. YouTube). This

accessibility in learning ensures that the assembly process is manageable, thereby facilitating broader adoption and utilization in research settings.

R5) *"The community of researchers applying the perceptual crossing paradigm is not especially large. What applications might this device have outside of perceptual crossing?"*

We primarily envision its broader utility in studies of real-time embodied social interaction, including hyperscanning approaches. We now highlight this utility as follows:

"The PCD offers potential applications beyond its initial scope as well. Notably, in the field of hyperscanning, where each study typically employs its own unique system for data collection, the lack of standardization complicates the replication and comparison of data across studies. The PCD could provide a standardized solution that enhances data uniformity, thus improving community-wide data sharing and validation within the hyperscanning field." (Line 386 – 391)

This application leads to replicability of findings of collective behavior in the community. Furthermore, the PCD holds significant potential for advancing research into social disorders, thereby opening a new avenue for contributions to mental health studies, for example, Zapata-Fonseca et al. (2018) and Zapata-Fonseca et al. (2024). These applications not only extend the utility of the PCD but also align with the growing need for consistent methodologies and collaborative efforts in neuroscience research.

Zapata-Fonseca L, Froese T, Schilbach L, Vogeley K, Timmermans B. Sensitivity to Social Contingency in Adults with High-Functioning Autism during Computer-Mediated Embodied Interaction. Behavioral Sciences. 2018;8(2). doi:10.3390/bs8020022.

Zapata-Fonseca L, Belhadi A, Leenen I, Sangati F, Fossion R, Fuchs T, et al.. Toward a second-person approach to investigating schizophrenia: (mis)perceiving others in the perceptual crossing experiment?; 2024. Available from: osf.io/preprints/psyarxiv/249je.

Minor Comments

R6) *"Lines 6 – 8: expand on the difference made by engaging, real-time social interactions and why this difference is meaningful/pressing for studies of social behavior and social cognition."*

The PCD facilitates dynamic interactions between two individuals, which is crucial because dyadic interactions elicit distinct brain activity compared to passive observation or non-interactive social observation. We now expand on this crucial point as follows:

"Second-person neuroscience studies have demonstrated that even the brain activity is distinctive when comparing dyadic interactions with passive and social observations [1]. Accordingly, it has been argued that every experimental setup assessing social interaction should include at least two people dynamically interacting, a shared context in which participants can both feel engaged, and data collection and analysis at the dyadic level [1]. " (Line 6 – 11)

[1] Schilbach L, Timmermans B, Reddy V, Costall A, Bente G, Schlicht T, et al. Toward a second-person neuroscience1. Behavioral and brain sciences. 2013;36(4):393–414.

R7) *"Lines 9 – 12: It seems worthwhile to briefly state the broad goal of the PCE at this point, in addition to the format of the experiment. I note that the authors describe the task on lines 30 – 31, but making the broad goal clear early (and later providing additional details, which the authors have done) will help readers not only understand the context of the PCE but also the contribution of the PCD to the field more broadly."*

We now clarify that, in the PCE, we are assessing the social interaction between participants:

"Accordingly, it has been argued that every experimental setup assessing social interaction should include at least two people dynamically interacting, a shared context in which participants can both feel engaged, and data collection and analysis at the dyadic level [1]. One paradigm that fulfills such requirements is the Perceptual Crossing Experiment (PCE). In the PCE, pairs of participants interact through a minimalistic interface based on a continuous sensorimotor feedback loop consisting of hand-controlled movements and tactile feedback patterns (See Fig. 1)." (Line 8 – 15)

[1] Schilbach L, Timmermans B, Reddy V, Costall A, Bente G, Schlicht T, et al. Toward a second-person neuroscience1. Behavioral and brain sciences. 2013;36(4):393–414.

R8) *"Lines 19 – 20: The authors say the new PCD "provides a novel framework." Is the framework simply the integration of physiological data with a sensorimotor interaction task? The nature of the framework and what about it is novel is unclear to me."*

Yes, an essential advance is that the framework of the PCD is designed to be compatible with hyperscanning techniques, which allows for the simultaneous recording of brain activity from multiple participants. The novelty of the PCD primarily resides in its design feature that offers a 1-to-1 augmented space, the transparency of the device specs, and its wide availability to users in the public domain. This is further explained in the following sentences:

"The introduction of the Perceptual Crossing Device (PCD) in this study establishes a novel framework aimed at supplying a transparent, publicly available device for researchers working with haptics interested in the social domain to examine sensorimotor dyadic interactions in real-time. The novelty of the device enhances user engagement through an augmented space that provides immediate feedback on user movements and incorporates extensive haptic feedback, while the framework allows for compatibility with hyperscanning equipment to record the brain activity of participants concurrently. These features are designed to facilitate a more comprehensive understanding of embodied interactions by integrating the dynamics of two interacting brains." (Line 24 – 33)

R9) *"Please report how the (absence of) delay in sensory feedback compares to previous iterations of the PCD."*

The absence of delay in sensory feedback in our current model marks a significant enhancement. We now make it clear in the following sentences:

"Furthermore, unlike earlier versions of the PCD, details on validation and replicability have not been publicly disclosed until now. We are the first to provide such information, offering valuable resources for researchers, behavioral scientists, cognitive scientists, cognitive neuroscientists, and psychologists." (Line 84-87)

What distinguishes our device is not only its enhanced performance but also our commitment to transparency. We provide comprehensive specifications and have made the device completely open source. This approach not only facilitates more accurate comparisons with other research but also significantly contributes to the field by enhancing the reproducibility and collaborative potential of future studies.

R10) *"The authors provide instructions for 3D printing materials through an external company but also include CAD files for 3D printing. Do these CAD files contain all the necessary information to 3D print all parts oneself?"*

The CAD files provided include all necessary specifications for 3D printing the device components.

R11) *"In several instances throughout the paper, what should be open quotation marks (') are actually end quotation marks ('). In LaTeX, an open quote is achieved with `."*

The improperly placed quotation marks have been corrected.

R12) *"Line 223: typo ('more specific the precise recording of button presses')"*

The typo has been corrected.

R13) *"The authors note that the tactile stimulation has a mean latency of 16.2 ms and the audio stimulation has a mean latency of 3.2 ms. Given that PCE largely has a psychologically-oriented audience of researchers, it would be worthwhile to include a reference confirming that both of these latencies are shorter than a latency that is detectable by or influential to humans."*

We have added a reference to substantiate that the latency in our device's audio and tactile feedback is below the threshold detectable by humans.

"This temporal precision is critical for researchers and practitioners conducting trials using the PCD and shows the real-time feedback to be shorter than the average latency detectable by humans for haptic and audio feedback at 205 ms and 245 ms respectively [20]." (Line 347 – 350)

[20] Peon AR, Prattichizzo D. Reaction times to constraint violation in haptics: comparing vibration, visual and audio stimuli. In: 2013 World Haptics Conference (WHC); 2013. p. 657–661.

R14) *"In-text caption for Figure 13 presumably has a typo. I assume it should say auditory feedback?"*

You are right, the typo has been corrected.

R15) *"The authors note a key limitation is the rotary encoder's resolution. It would be helpful to comment on the extent to which this resolution is detectable by humans or could possibly influence behavioral or psychophysiological data."*

We have looked deeper in this comment and have addressed concerns regarding the rotary encoder's resolution as follows:

"A limitation to consider is the encoder's resolution, which is specified at 1024 pulse/rotation. Given that a complete revolution in the virtual environment equates to 600 units, it can be deduced that each pulse from the encoder corresponds to a movement of approximately 0.588 units (0.35^o) in the virtual space. Specifically regarding the operational precision of our device, the circumference of the spinning handle's field of movement on the controller measures 400.55 mm. With the encoder configured to 1024 pulses/rotation, each pulse translates to approximately 0.39 mm along the circumference, which constitutes roughly 0.1% of the total circumference. Given this precision, we assessed that, although more accurate encoders are available, the resolution provided by our current encoder is sufficiently precise for detecting human movements in the experimental setup." (Line 321 – 331)

R16) *"Low resolution for figures 3 and 9. Could the authors provide higher resolution images?"*

Figures 3 and 9 have been updated with higher resolution images to enhance clarity.

R17) *"Could the authors discuss the longevity of the PCD? What is involved in device maintenance? To what extent and at what rate might the signal quality degrade over time?"*

We have expanded on the maintenance of the device to clarify that upkeep primarily involves ensuring screws are tightly secured, maintaining the structural integrity of the device:

"The primary maintenance activity involves ensuring that the screws securing the centrally positioned B-type timing pulley (GPA40GT2060-B-P10) and the 3D printed coupling component, which links the B-type pulley to the slip ring, are adequately tightened. Furthermore, it is crucial

to ensure that the LRA motor within the spinning handle is securely affixed to the inside of the handle to allow proper haptic feedback." (Line 241 – 246)

Overall

R18) *"Has the statistical analysis been performed appropriately and rigorously? No."*

We did not include statistical analysis as it did not seem necessary for the technical objectives of our contribution.

Thank you for the thoughtful review of the PCD technical report, which has helped us to clarify the key elements of our contribution.
